# Transparency – a patient-centric view on radiopharmaceutical extravasations

**DOI:** 10.3389/fnume.2023.1127692

**Published:** 2023-02-27

**Authors:** Pam Kohl

**Affiliations:** Independent Researcher, Raleigh, NC, United States

**Keywords:** patient safety, infiltrations, extravasations, healthcare transparency, patients, radiopharmaceutical

## Abstract

Most radiopharmaceuticals are intravenously administered during nuclear medicine imaging or therapy procedures. When a nuclear medicine clinician delivers some or all of a radioactive drug into a patient's healthy tissue rather than the vein as intended, a patient experiences an extravasation. Radiopharmaceutical extravasations provide zero patient benefit and considerable potential downsides, depending on the severity of the extravasations. What nuclear medicine patients want and need regarding the administration of radiopharmaceuticals is transparency. And yet in the year 2023, little transparency exists regarding these extravasations. From the patient perspective, transparency regarding extravasations is essential to improving care, ensuring radiation protection, reducing health inequities, and untangling the deeply disturbing and irregular relationship between the nuclear medicine community and their regulating body, The U.S. Nuclear Regulatory Commission. Transparency is also critical to help address many other questions regarding radiopharmaceutical extravasations.

## Introduction

I spent the last 10 years of my career working for one of the largest breast cancer advocacy organizations in the world. In this role, I learned how important it is for patients to advocate for themselves. I also saw the positive role that nuclear medicine plays in cancer care. When I was diagnosed with recurrence of my breast cancer, I advocated for a PET/CT scan even though my physician said it was not needed. As a result of my advocacy, we learned that my breast cancer had metastasized. Without nuclear medicine, without that PET/CT scan, my metastatic breast cancer (MBC) diagnosis would have likely come too late, meaning I would probably not be authoring this article now.

MBC has no cure and living with this disease is extremely stressful. I will be in treatment for the rest of my life and appropriate treatments extend my life. I will rely even more on nuclear medicine and will have imaging procedures every three months to monitor the progression of my disease. Accurate images are critical to my treatment plan and my life. Knowing that my nuclear medicine images are accurate helps ease my anxiety. An inaccurate image is not good for my care.

**Figure 1:**
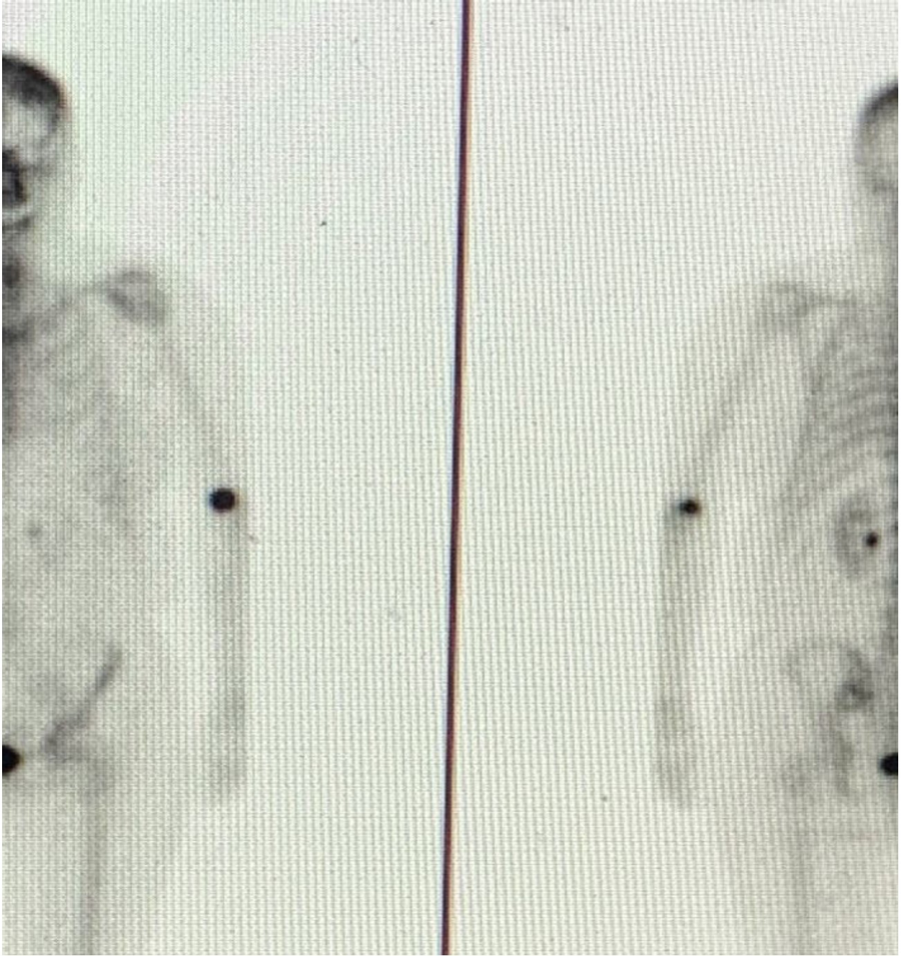
Anterior and posterior MDP images of extravasation.

During my early-stage cancer treatment and the six years of my metastatic journey, I have received over 40 nuclear medicine procedures at a world-renowned hospital. During this journey, many different technologists have administered my radiopharmaceuticals and I have witnessed varying skills, techniques, and tools. Based on numerous administration experiences and because of my awareness of the extravasation issue through my association with the coalition, Patients for Safer Nuclear Medicine, I suspected that I had been extravasated during a routine MDP bone scan 18 months ago. My technologist was unconvinced. She assured me the radioactive technetium and MDP had been administered properly. I fiercely pressed for evidence and finally succeeded in having my arm imaged (Figure 1). The technologist was shocked to find that she had extravasated me. But shock did not equal action. In fact, there were no mitigation efforts. Instead, I was left with the majority of the 22 mCi of administered technetium deposited in my tissue instead of my vein. And even though I did not develop visible symptoms on my skin, I suffered from pain at the injection site for days and weeks later. To this day, the area routinely aches.

My goal is to share the patient perspective about the need for transparency regarding radiopharmaceutical extravasations. Much of this perspective has been informed from my personal experience, but it also reflects what I have learned from other patients and members of the Patients for Safer Nuclear Medicine coalition. This perspective on transparency covers patient care, radiation protection, health inequity, and the regulatory framework in the United States guiding the use of medical isotopes. It also reflects many additional questions related to radiopharmaceutical extravasations that highlight the need for more transparency.

### Patient care

As patients, we want to know, as is our right, when a large radiopharmaceutical extravasation has been severe enough that our diagnostic images or delivery of our therapy may have been compromised. For patients, it is essential that nuclear medicine clinicians are aware when large extravasations occur, measure them, quantitatively assess the impact to our procedure, and communicate this information to us and to our treating physicians. We understand that not all procedures go as planned. But when they don't, we expect and need to be told, since these procedures directly or indirectly affect our care (1–8). If we need additional therapy delivered or if we need to repeat an imaging procedure because of an extravasation, we expect this information to be shared with us and that actions be taken to prevent a repeat extravasation.

I do not truly understand the physics of nuclear medicine procedures. Nor do most patients. But clinicians understand. Clinicians and physicists know that depositing ionizing radiation accidentally into a patient's tissue is not appropriate, yet very few nuclear medicine centers proactively monitor radiopharmaceutical administrations for extravasations or measure the radiation that is inadvertently deposited into tissue. But when extravasations happen, patients want to know how much accidental radiation they have received to their arm tissue. They want to know that their clinicians are well trained in immediate mitigation techniques and will take appropriate and steps to minimize the radiation dose to their tissue because of this misadministration. Patients want to know what symptoms to expect and when they might develop. When my extravasation occurred, no mitigation took place to minimize the radiation dose to my tissue and no communication regarding what I should expect ever occurred.

I have spoken with many patients about this issue. While the radiation effects to our arm tissue are important, most older patients are more concerned about the implications to our procedure and follow-on treatments. From my time working with PSNM, I know that clinicians need specific information about the extravasation to best estimate the effect to our procedure. They need to know the amount of the activity left in the arm at imaging time, how the patient's body reabsorbed the accidental radiation in the tissue, and how this uptake delay affected any quantification measurements of their procedures. My care team is concerned about the metabolic uptake of my tumors from one scan to the next. The amount of radiation still in my arm, in my vascular system, and lymphatic system at time of imaging is important. Yet, in my extravasation case no measurement took place either.

Extravasated patients also want to know of other potential effects or important precautions. If a large amount of radioactivity is still left in the patient's arm, what are the implications for exposing others after the procedure is over? What are long-term implications of radiation to the patient's healthy tissue?

A leading nuclear medicine physician in my home state of North Carolina is on the written record that patients already have a lot to worry about, implying that telling them that they have experienced a large extravasation would further alarm them. Another nuclear medicine physician lectured me on why I did not need to worry about extravasations. Patients do not want to be patronized or told to not worry about these accidents. Patients want information. They want it documented in their medical records. They want to make sure that their primary caregivers who should know they were extravasated are told. Patients want their future nuclear medicine clinicians to know their history of extravasation so clinicians take extra precaution to avoid extravasating the patient again. It is inconceivable that in the year 2023 transparency for extravasated patients is not the standard of care.

### Radiation protection

The U.S. Nuclear Regulatory Commission (NRC) has regulations regarding the use of medical isotopes. They are in place to ensure the security of isotopes and their proper use in United States patients. When an isotope is mishandled due to human error, lack of training, or lack of quality processes, and results in the inadvertent exposure to patients that exceeds a certain absorbed dose, regulations require these cases be reported. The goal of this reporting is to ensure that lessons learned from these cases are shared with other nuclear medicine centers so that future patients are protected. Reported cases are also shared publicly.

Since 1980, the NRC has exempted all extravasations from reporting (9), no matter how much accidental radiation patients receive. Ironically, if a technologist spills a radioisotope on a patient, they will clean it up, estimate the radiation exposure to the patient's skin and tissue, report it if it exceeds the NRC limit, and perform a root cause analysis. Yet if the same technologist extravasates or spills the same radioisotope inside the patient's body, into their tissue, nothing needs to be done. Nothing needs to be reported, even if the radiation dose to the patient's skin and tissue is twice as much as the spill on the patient. Even if it is 100 times more than the spill on the patient. The NRC internal exemption policy created this alarming situation. When I share this story with other patients, they shake their head in disbelief.

This policy was based on the belief that an extravasation is “virtually impossible to avoid” (9). Nothing could be farther from the truth. Evidence from other similar IV practices and from the literature is clear. Chemotherapy and Contrast CT extravasation rates are very low (10, 11), indicating that radiopharmaceutical extravasations can be almost completely eliminated as well. Nuclear medicine centers already know which of their technologists routinely extravasate patients. They know which technologist to avoid if their family members need care. And patients who routinely experience nuclear medicine procedures also know which technologist are best at administering radiopharmaceuticals. I now know to only use the hospital's IV team to set up my IV access for nuclear medicine procedures. It is a luxury that my hospital has an IV team. And ever since my extravasation, it is a luxury I will continue to use. Most patients receive their care from providers that don't offer such an option.

If the 1980 incorrect reporting exemption did not exist, patients would have more information about providers. Transparency regarding which centers routinely extravasate helps patients make better decisions about where to seek imaging or therapy procedures. Centers that do not extravasate would have nothing to report. Centers that accidentally expose patients routinely to high doses of radiation would be reporting frequently. These centers would come under closer scrutiny that would lead to efforts to improve. Without a spotlight on this issue, with no requirement to report large accidental exposures to patient tissues, nuclear medicine centers will continue to operate as they always have. Most centers will continue to avoid making the necessary investments to fix their extravasation issues. Transparency will lead to improved administrations and improved radiation protection for patients.

### Healthcare inequities

Many nuclear medicine professionals are on the record in their conversations with the NRC that transparent reporting of extravasations may bankrupt nuclear medicine providers and limit which centers are able to perform these procedures. They claim that centers will have to spend inordinate sums to provide training and to purchase IV access tools and monitoring technology. And centers who have a bad reputation for extravasations may have to close down.

Patients have a different perspective. We know that these centers make substantial investments in all other quality control aspects of nuclear medicine procedures. The list of quality activities to ensure that the right drug and the right radiation dose gets to the right patient at the right time is impressive. Patients do not believe that adding one more quality check will put centers out of business. Patients are also clear that we, along with insurance companies, pay for these procedures. Patients do not want to pay for procedures that have been compromised by large extravasations. Patients also think that centers that don't improve *should* go out of business; patients do not want to have their nuclear medicine procedures performed at centers that routinely make mistakes handling medical isotopes.

Regulations that are consistently applied across all providers will reduce healthcare inequities. It is not fair to patients of lower economic means if only well-funded private providers voluntarily decide to address extravasations. Regulations will incent all centers to make the required investments to minimize extravasations.

### The NRC and medical societies

The Advisory Committee on the Medical Uses of Isotopes (ACMUI) provides advice to the NRC regarding medical issues. The ACMUI is almost entirely comprised of professionals who are affected by NRC regulations. Many are leaders from the medical societies, like the Society of Nuclear Medicine and Molecular Imaging (SNMMI). Over the past 42 years, the ACMUI and SNMMI have worked with the NRC medical staff to provide advice that ensures that radiopharmaceutical extravasations remain exempted from reporting.

In a public phone conference with NRC on the topic of extravasations, Dr. Carol Marcus, a former member of the ACMUI, noted how she worked to retain the exemption in the early 1990 s. In 2008 and 2009, the ACMUI members in a meeting with NRC staff admitted that some diagnostic radiopharmaceutical extravasations could result in doses that easily exceeded reporting thresholds, and that these extravasations could be prevented by increasing training and improving venous access tools. They actually stated that the reason they wanted to retain the exemption was so they would not have to deal with the administrative burden of reporting large extravasations (12, 13). Despite hearing that extravasations were not virtually impossible to avoid, and patients were receiving very high doses to their tissue, the NRC medical staff looked the other way and allowed the exemption to continue. As a patient reading the meeting transcripts, I was extremely disturbed by the ACMUI members' behavior and the NRC medical staff's willingness to ignore this patient radiation protection issue.

Most recently, the NRC has been considering a petition for rulemaking^1^ to remove the reporting exemption and treat extravasations no differently than any other medical event. Medical societies, and the ACMUI, aware that the exemption is being threatened again have misled NRC with misinterpretation of certain publications (7), confusing rates of injuries with rates of reported injuries. They have also provided a suggested reporting option that will minimize reporting. This option was adopted by the NRC medical staff as their recommendation to the NRC Commissioners on how to address the petition. The medical society and NRC medical staff recommendation suggests that NRC should require patients be injured, return to the center that caused their injury, and get a physician to then report the extravasation voluntarily to the NRC.

This exact option had previously been considered and dismissed by the Commissioners 40 + years ago for many legitimate reasons (9). The jointly proposed option by medical societies and the NRC medical staff shifts the responsibility for the proper use of medical use of a radioactive isotope from a trained clinician to the patient. It requires that patients be injured before reporting, which is not a requirement for any other NRC reportable event. In fact, the NRC website^2^ and the medical event form instructions state that patient harm is not a requirement for medical event reporting^3^. The reporting requirement is specifically designed to highlight facilities that *may have a potential problem* in the proper handling of medical isotopes. Facilities that routinely and accidentally expose patients' healthy tissue to high doses of radiation are exactly the facilities that the NRC needs to investigate.

This patient injury option makes no sense in so many other ways. Without providers monitoring for extravasations, patients will not be told they have been extravasated. Also, the injuries can come weeks to months to years later. It is not appropriate to expect that patients would connect the injury with the procedure, especially if they were not informed they had been extravasated. And without monitoring for extravasations, providers cannot provide patients with immediate mitigation efforts. If patients are not told they have been extravasated but do feel or see symptoms months later and just happen to suspect extravasation, they would be forced to try and schedule an office visit that the patient would have to pay for. As patients, we know it can be many weeks before we can even see a doctor. What if our symptoms lessen during this period? What if we receive our procedure in a remote location, but the physician we must see to assess our injury is at a central hospital location? Now we must pay for a visit and perhaps travel many hours.

Finally, patient injury as the reporting criterion further increases healthcare inequities. During a recent video call with the Chairman of the NRC, two African American patients pointed out that patients of color would be extremely hesitant to return to a center and report a patient injury. Requiring patient injury rather than the quantifiable threshold used for reporting all other medical events to the NRC would ensure that extravasations remain essentially unreported and would do nothing to reduce healthcare inequities.

During the submission process of this article, the NRC ruled on the petition. I have read the Federal Register announcement dated December 30, 2022^4^. The NRC accepted the petition and is moving the incorrect exemption policy issue into rulemaking. In essence, the NRC agreed with nearly every aspect of the petition. They did not deny the science, or the clinical evidence provided. Yet, the Commissioners have adopted the medical staff's recommendation for patient injury as the reporting criterion^5^. To avoid having to address the actual occurrence of extravasations and to appease the physicians they regulate, Commissioners have ignored all the science and their own policies and past comments and supported patient injury as a reporting criterion. The following two paragraphs in a commissioner's explanation^6^ for their support of the patient injury recommendation provides clarity about the absurdity of their conclusion.

“The purpose of medical event reporting to the NRC is to both collect operating experience and ensure that licensed activities are conducted safely. Importantly, a medical event may indicate a potential problem in a medical facility's use of radioactive material, but it does not necessarily mean that a patient has been harmed. Rather, the information is used to assess trends, identify generic issues or concerns, and recognize and respond to the inadequacy or unreliability of specific equipment or procedures.

While some level of extravasation may, in fact, be virtually impossible to avoid in any intravenous or intra-arterial injection, there are medical techniques and tools in place to help ensure that radiopharmaceutical injections reach the intended target organ or organs. When these procedures fail to a degree that they result in unintended radiation injury, then it is reasonable and warranted for the NRC to understand the circumstances around these injuries and consider the factors that may prevent them, such as training, additional tools, and mitigation measures.”

In his first paragraph, the commissioner explains why using the existing dose threshold reporting criterion, rather than patient injury, for medical event is necessary. In his second paragraph, he states that the NRC should only be concerned about extravasation when patients are injured. The commissioners' decision is an embarrassment to the NRC and the radiation protection of patients.

Patients want transparency in the regulatory process that is responsible for providing patients with radiation protection. The relationship between the medical societies and the NRC is incestuous. The medical staff recommendation and endorsement by NRC Commissioners are of great concern for patients. Even the current patient advocate on the ACMUI has strong ties to the SNMMI. These medical societies and members of the ACMUI have inherent conflicts of interest and have been disingenuous with the NRC medical staff and the Commissioners. Unfortunately this hurts patients (1). In a recent op-ed, Dr. Dan Fass claimed the NRC was guilty of regulatory capture^7^. Dr. Fass suggested a possible similar relationship would be like if Boeing and Airbus were allowed to craft FAA regulations. I liken the situation to the proverbial fox guarding the hen house. In fact, in North Carolina, this is exactly what happens. The North Carolina Radiation Protection Commission is comprised of nuclear medicine professionals. One of their responsibilities is to draft the regulations that govern their own profession.

## Discussion

The lack of transparency regarding radiopharmaceutical extravasations needs to be addressed. In the United States alone, over 30 million radiopharmaceuticals are administered every year. And now, new radioactive drugs with very high levels of radioactivity are being introduced to address cancers that have previously resisted treatment. Nuclear medicine procedures are increasingly being used for diagnosing and assessing treatments of neurological diseases. And the most prevalently performed nuclear medicine procedures continue to be cardiology procedures. In these procedures that produce images of the heart, no one views the injection site for evidence of extravasations. Yet, with all of these procedures and the growing use of new therapeutics, we do not know how many procedures are being compromised by large extravasations.

No one would argue that it is wrong when a patient on a hospital's general floor only receives some of their medication. No medical professional would ignore a patient who experiences a chemotherapy extravasation. They would work to mitigate the effects of the drug on the healthy tissue. Yet ionizing radiation accidently deposited in tissue has been and will continue to be ignored in the United States.

Many questions remain unanswered. How often do technologists extravasate? Are some technologists better than others? Are some centers better than others? Are patients of color more often extravasated than other patients? What are best practices for administering radiopharmaceuticals? What specific training and which tools are needed to get technologists to the same skill level as the IV team members? Are some tools better than others for gaining venous access or administering radiopharmaceuticals? What is a safe amount of radioactivity that can be left in tissue? Does every nuclear medicine center have a protocol on how to address a radiopharmaceutical extravasation? How do they measure the amount of activity left in the arm? How does the nuclear medicine center decide on which extravasated procedures get repeated? How many of extravasated procedures are repeated? Who pays for them? How many should be repeated? Are patients and their treating physicians informed when patients are extravasated? Is it ok for a grandmother with an extensive extravasation of a gamma emitting radioisotope with a long half-life to go home and babysit? Is it ok for her to hold her infant grandbaby in her arms with their head resting on the grandmother's extravasation site? And if she is not told, how would she know not to hold her grandbaby? And what of the longer-term implications of large extravasations for younger patients? Isn't it known that some level of radiation exposure can lead to cancer later? Who is tracking these extravasated patients for cancer? If radiopharmaceutical administrations are not prospectively monitored, and extravasations not identified, not measured, and not documented, how would we know what are the long-term effects for younger patients?

The extravasation of a radiopharmaceutical is clearly a misadministration of a drug. It is a medical error that can have consequences for patients. Without complete transparency, these questions and many others will remain unanswered. That is unacceptable to patients.

## Data Availability

The original contributions presented in the study are included in the article/Supplementary Material, further inquiries can be directed to the corresponding author.

## Footnotes

^1^ https://www.regulations.gov/docket/NRC-2020-0141

^2^ https://www.nrc.gov/reading-rm/doc-collections/fact-sheets/risks-assoc-medical-events.html

^3^ https://www.nrc.gov/reading-rm/doc-collections/event-status/event/2021/20210419en.html#en55186

^4^ https://www.federalregister.gov/documents/2022/12/30/2022-28356/reporting-nuclear-medicine-injection-extravasations-as-medical-events?utm_source=federalregister.gov&utm_medium=email&utm_campaign=subscription±mailing±list

^5^ https://adamswebsearch2.nrc.gov/webSearch2/main.jsp?AccessionNumber=ML22346A115

^6^ https://adamswebsearch2.nrc.gov/webSearch2/main.jsp?AccessionNumber=ML22321A145

^7^ https://www.statnews.com/2021/12/27/nuclear-regulatory-commission-foot-dragging-irradiation-rule-suggests-regulatory-capture/
